# Minimally invasive ureteroplasty with lingual mucosal graft for complex ureteral stricture: analysis of surgical and patient-reported outcomes

**DOI:** 10.1590/S1677-5538.IBJU.2023.0393

**Published:** 2024-03-18

**Authors:** Xiang Wang, Chang Meng, Derun Li, Yicen Ying, Yunke Ma, Shubo Fan, Xinfei Li, Kunlin Yang, Bing Wang, Hua Guan, Peng Zhang, Jing Liu, Chen Huang, Hongjian Zhu, Kai Zhang, Liqun Zhou, Zhihua Li, Xuesong Li

**Affiliations:** 1 Peking University Institute of Urology National Urological Cancer Centre Beijing China Department of Urology, Peking University First Hospital, Institute of Urology, Peking University, National Urological Cancer Centre, Beijing, China; 2 Peking University Peking University First Hospital Department of Nursing Beijing China Department of Nursing, Peking University First Hospital, Peking University, Beijing, China; 3 Emergency General Hospital Department of Urology Beijing China Department of Urology, Emergency General Hospital, Beijing, China; 4 Beijing Jiangong Hospital Department of Urology Beijing China Department of Urology, Beijing Jiangong Hospital, Beijing, China

**Keywords:** Minimally Invasive Surgical Procedures, Plastic Surgery Procedures, Quality of Life

## Abstract

**Objective:**

To evaluate objective treatment efficacy and safety, and subjective patient-reported outcomes in patients with complex ureteral strictures (US) undergoing minimally invasive lingual mucosal graft ureteroplasty (LMGU).

**Materials and Methods:**

We prospectively enrolled patients underwent robotic or laparoscopic LMGU between May 2020 and July 2022. Clinical success was defined as symptom-free and no radiographic evidence of re-obstruction. Patient-reported outcomes, including health-related quality of life (HRQoL), mental health status and oral health-related quality of life (OHRQoL), were longitudinally evaluated before surgery, 6 and 12 months postoperatively.

**Results:**

Overall, 41 consecutive patients were included. All procedures were performed successfully with 32 patients in robotic approach and 9 in laparoscopic. Forty (97.56%) patients achieved clinical success during the median follow-up of 29 (range 15-41) months. Although patients with complex US experienced poor baseline HRQoL, there was a remarkable improvement following LMGU. Specifically, the 6-month and 12-month postoperative scores were significantly improved compared to the baseline (p < 0.05) in most domains. Twenty-eight (68.3%) and 31 (75.6%) patients had anxiety and depression symptoms before surgery, respectively. However, no significant decrease in the incidence of these symptoms was observed postoperatively. Moreover, there was no significant deterioration of OHRQoL at 6 months and 12 months postoperatively when compared to the baseline.

**Conclusions:**

LMGU is a safe and efficient procedure for complex ureteral reconstruction that significantly improves patient-reported HRQoL without compromising OHRQoL. Assessing patients’ quality of life enables us to monitor postoperative recovery and progress, which should be considered as one of the criteria for surgical success.

## INTRODUCTION

The treatment of long proximal ureteral stricture (US) remains a challenge for urologists. Since the first case of laparoscopic lingual mucosal graft ureteroplasty (LMGU) was introduced by Li et al. in 2015 (1), it received extensive attention as an efficient way to avoid ileal ureter replacement or renal autotransplantation for ureteral reconstruction (2). Recently, Yang et al. reported the technique and initial experience of robotic LMGU with encouraging perioperative and medium-term follow-up outcome (3). Then, Liang et al. shared their 6 years of experience with 41 cases of LMGU (4). However, the efficacy and durability of LMGU remains to be validated.

When surgical treatment is available, counseling and education about surgical effectiveness, complications and postoperative recovery are necessary to facilitate patient decision making (5). While surgical effectiveness can be efficiently evaluated by experienced surgeons through postoperative examinations and radiographic results, one of the most important goals of any surgical interventions is to improve patients’ quality of life. Measuring preoperative and postoperative changes of HRQoL provides another dimension to assess the outcomes of surgical treatment. However, no previous study has focused on the health-related quality of life (HRQoL) and mental health status in patients undergoing minimally invasive LMGU. Moreover, the morbidity of lingual mucosal graft (LMG) harvesting and postoperative oral health-related quality of life (OHRQoL) recovery has not been well documented.

Herein, we prospectively recruited a cohort of patients to evaluate the objective surgical efficacy and safety of minimally invasive LMGU. Moreover, we also longitudinally evaluated the changes of subjective patient-reported outcomes, including HRQoL, mental health status and OHRQoL before and 12 months following minimally invasive LMGU.

## MATERIALS AND METHODS

### Study Population

Patients with complex US undergoing robotic or laparoscopic LMGU between May 2020 and July 2022 at three centers were prospectively enrolled. The indications for surgery were defined as persistent clinical symptoms, deteriorated renal function and obstruction demonstrated by image examinations. LMGU was adopted due to the unsuitability for simple anastomosis, renal pelvic flap and appendiceal flap, according to our substantial experience (3, 6). All procedures were performed by one experienced surgeon.

Inclusion criteria were as follows: [1] aged 18-75 years; [2] diagnosed with complex US undergoing robotic or laparoscopic LMGU, [3] ability to read and write in Chinese, and [4] willingness to adhere to scheduled follow-up. Exclusion criteria included: [1] refusal or inability to sign informed consent, [2] simultaneously underwent other reconstructive procedures, [3] illiteracy, [4] disabilities, or [5] cognition or behavioral impairment. This study followed the Strengthening the Reporting of Observational Studies in Epidemiology (STROBE) reporting guideline. Informed consent was obtained from all patients and this study was approved by the Ethics Committee of the Peking University First Hospital (approval number: 2019SR134).

### Data collection

The patients’ demographics, perioperative and follow-up results were prospectively collected in our Reconstruction of Urinary Tract: Technology, Epidemiology and Result (RECUTTER) database. The surgical technique has been reported in our previous researches (3, 7-9). Clinical success was defined as symptom-free and no obstruction on radiographic evaluation. We used the MOS 36-item health survey (SF-36), the hospital anxiety and depression scale (HADS), and the Oral Health Impact Profile-14 (OHIP-14) to measure HRQoL, mental health, and OHRQoL, respectively. Patients received and completed the questionnaires by self-administration the morning before the operation, 6 months and 12 months afterwards.

### Quality of Life Instrument

MOS 36-item health survey (SF-36)

The generic aspects of HRQoL were assessed using the Chinese version of RAND 36-Item Health survey 1.0 (SF-36), which has been adequately validated in Chinese population and widely used (10) (see Appendix-1 click here). The SF-36 consists of 36 multiple-choice questions summarized into eight distinct domains: physical function (PF), role limitations due to physical health problems (RP), bodily pain (BP), and general health perception (GH), vitality (VT), social function (SF), role limitations due to emotional problems (RE), and mental health (MH). Each domain consists of 2 to 10 items, evaluated on a 2–6-point Likert scale. Scores for each domain range from 0 to 100. Higher scores indicate higher function or well-being. The eight domains can be further grouped into two summary scores: physical component summary (PCS) and mental component summary (MCS) (10).

### The hospital anxiety and depression scale (HADS)

The Chinese version of hospital anxiety and depression scale (HADS) was used to measure the level of anxiety and depression in participants. The scale has been validated in Chinese population and is widely used to evaluate anxiety and depression with well screening utility(11). The HADS, a 14-item questionnaire, is comprised by two subscales: anxiety (7 items) and depression (7 items). Each item is evaluated on a 4-point Likert scale. For each subscale, the scores range from 0 to 21. Higher scores indicate higher levels of anxiety/depression. In the present study, the anxiety/depression scores of HADS were divided into two categories: non-anxiety/depression (0-7) and HADS-anxiety/HADS-depression (8-21).

### Oral Health Impact Profile-14 (OHIP-14)

The OHRQoL was measured by the Chinese version of Oral Health Impact Profile-14 (OHIP-14) (12). The OHIP-14 questionnaire consists of 14 items structured in seven domains, namely functional limitations, physical disability, psychological disability, physical pain, psychological discomfort, social disability, and handicap. Each item scores on a 5-point Likert scale. The scores of OHIP-14 range from 0 to 56. Lower scores indicate better OHRQoL.

### Statistical Analysis

Normally distributed continuous variables were reported as the mean and standard deviation (SD), while non-normally distributed continuous variables were presented as the median and range. Categorical variables were described as frequencies and proportions. The HRQoL of patients in this study was compared with that of the Chinese general population using Student's t test (5). Friedman test was used to compare changes in the scores along the follow-up. Wilcoxon signed-rank test was used to compare the postoperative scores to the preoperative values. Patients were considered to have significant improvement in HRQoL when the follow-up PCS/MCS scores improved more than 5 points compared with baseline. Statistical significance required two-tailed p values less than 0.05. All data were analyzed with the Statistical Package for Social Sciences v.16.0 (SPSS Inc, Chicago, IL, USA).

## RESULTS

**Figure 1:**
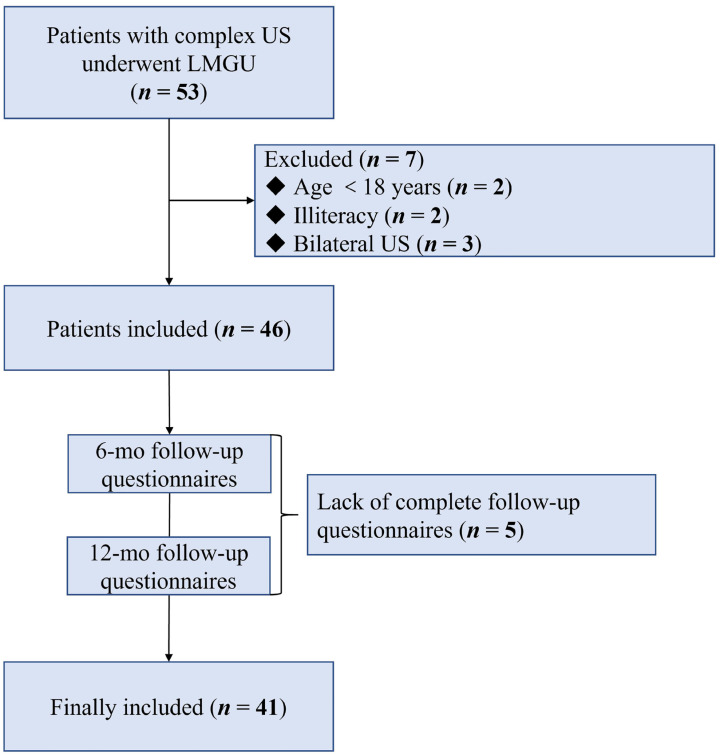
Diagram of the data collection process.

Overall, 53 patients diagnosed with complex US were proposed for reconstruction surgery with LMGU; 46 (86.8%) were included in our study, while 7 (13.2%) did not meet the eligibility criteria. Five of the 46 patients (10.9%) were excluded due to a lack of the complete follow-up questionnaires (Figure-1). Finally, the responses of the remaining 41 patients (89.1%) with complete follow-up data were evaluated.

Table-1 summarized the clinical and follow-up outcomes of the cohort. Nine female and 32 male patients with a median age of 33 years (21-67) were included. The etiology associated with stricture included ureteropelvic junction obstruction (15/41), ureteral calculi (23/41), ureteral polyps (1/41), laparoscopic renal cyst decortication (1/41), and laparoscopic adrenalectomy (1/41). The affected side was left side in 38 patients and right side in 3 patients. Of the 41 patients, 35 (85.37%) had proximal or ureteropelvic junction strictures, while 6 (14.63%) had mid-ureteral strictures. The mean stricture length was 3.50 cm. Twenty-one patients (51.22%) had a history of failed ureteral reconstruction.

**Table 1:** Clinical data and follow-up results of the cohort.

Variables		Value
Patients		41
**Gender, n (%)**		
	Male	32 (78.00)
	Female	9 (22.00)
Age, years, median (range)		33 (21-67)
BMI, kg/m^2^, mean (SD)		24.73 ± 3.70
**Etiology associated with stricture, n (%)**		
	UPJO	15 (36.58)
	Ureteral calculi	23 (56.09)
	Ureteral polyps	1 (2.43)
	Laparoscopic renal cyst decortication	1 (2.43)
	Laparoscopic adrenalectomy	1 (2.43)
**Affected side, n (%)**		
	Left	38 (92.68)
	Right	3 (7.32)
**Presenting symptoms, n (%)**		
	Symptomatic	20 (48.78)
	Incidental	21 (51.22)
**Stricture location, n (%)**		
	UPJ	14 (34.15)
	Proximal	21 (51.22)
	Middle	6 (14.63)
**Stricture length, cm, mean (SD)**		3.50 ± 1.56
Prior ureteral reconstruction, n (%)		21 (51.22)
**Type of surgical procedure, n (%)**		
Laparoscopic		9 (22.00)
Robotic assisted		32 (78.00)
**Surgical technique, n (%)**		
	Only ventral onlay	19 (46.34)
	Posteriorly augmented anastomosis with ventral onlay	22 (53.66)
Length of LMG, cm, median (range)		3.5 (2-7)
Width of LMG, cm, median (range)		1.5 (1-2)
Operative time, min, median (range)		189 (130-346)
EBL, mL, median (range)		50 (5-200)
Length of hospital, day, median (range)		6 (4-14)
Follow-up time, mo, median (range)		29 (15-41)
Clinical success, n (%)		40 (97.56)
**Complication rate, CD grade, n (%)**		7 (17.07)
	Tongue numbness (Grade I)	4 (9.75)
	Urinary tract infection (Grade II)	3 (7.32)

BMI = body mass index; SD = standard deviation; UPJO = ureteropelvic junction obstruction; UPJ = ureteropelvic junction; LMG = lingual mucosal graft; EBL = estimated blood loss; CD = Clavien-Dindo

All procedures were performed successfully with 32 patients using robotic approach and 9 the laparoscopic approach. Nineteen patients (46.34%) underwent posteriorly augmented anastomosis with LMG ventral onlay ureteroplasty, and the other 22 patients (53.66%) underwent only LMG ventral onlay ureteroplasty.

The median length and width of the LMGs were 3.5 cm (2-7) and 1.5 cm (1-2), respectively. The median operative time was 189 minutes (130-346), estimated blood loss was 50 mL (5-200), and postoperative hospital stay was 6 days (4-14).

No major postoperative complications occurred (grade III and IV). Four (9.75%) patients reported numbness of tongue within 1 month after surgery (grade I). Three (7.32%) patients developed urinary infection and respond well to antibiotic treatment (grade II). Clinical success was achieved in 40 (97.56%) patients during the median follow-up of 29 (15-41) months.

**Figure 2:**
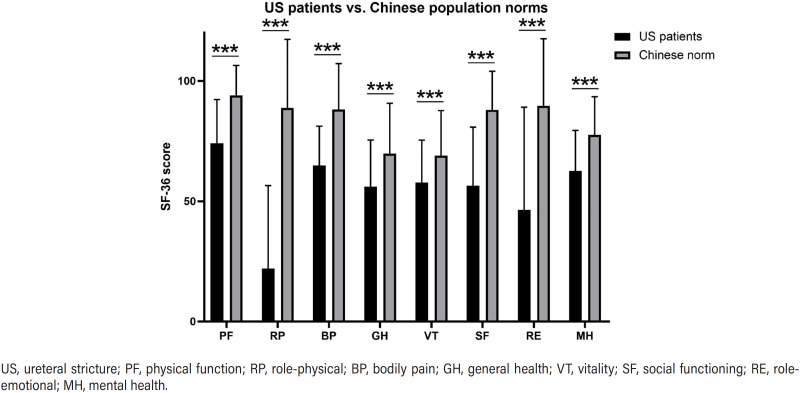
The comparison of SF-36 scores between patients with complex US in our study (n = 41) and the Chinese general populations (n = 3214). Error bars indicate standard deviation. Variables of significance (***p ≤ 0.001).

**Figure 3:**
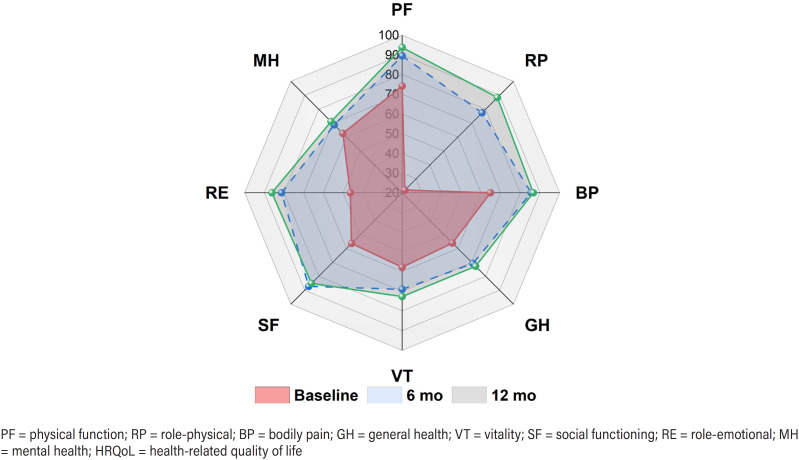
Longitudinal changes of mean scores in different SF-36 domains. Radar plots constructed to compare longitudinal changes of mean SF-36 scores in different domains. Higher scores indicated better HRQoL

Figure-2 outlined the mean scores for eight SF-36 domains. Patients with complex US presented significantly lower scores in all domains compared with the Chinese general population(5). Scores of each domain are shown in Table-S1. Radar plots were constructed to compare longitudinal changes of mean SF-36 scores in different domains (Figure-3). Comparisons between baseline and postoperative median scores of each SF-36 domains were conducted, revealing statistically significant modifications in all SF-36 domains (Table-2). Specifically, the 6-months and 12-months postoperative scores were significantly improved compared with the baseline values (p < 0.05) in all domains except MH. As for MH, there was significant improvement in 12-months postoperative scores but not at 6-months. At 12-months postoperatively, 35 (85.4%) and 26 (63.4%) patients achieved HRQoL success in PCS and MCS, respectively.

**Table 2:** HRQoL scores variation in the different domains during the follow-up (median and [IQR]).

Variable	Baseline	6 mo	12 mo	p#
PF	80 (62.5-95)	95 (90-97.5)	100 (95-100)	<0.001
*p* *	-	0.001	<0.001	-
RP	0 (0-25)	100 (62.5-100)	100 (100-100)	<0.001
*p* *	-	<0.001	<0.001	-
BP	64 (53-74)	84 (79-100)	84 (84-100)	<0.001
*p* *	-	<0.001	<0.001	-
GH	57 (45-72)	72 (55-92)	72 (57-91)	<0.001
*p* *	-	<0.001	<0.001	-
VT	60 (42.5-70)	70 (55-80)	80 (57.5-82.5)	<0.001
*p* *	-	0.033	<0.001	-
SF	50 (37.5-75)	87.5 (75-100)	100 (75-100)	<0.001
*p* *	-	<0.001	<0.001	-
RE	33.3 (0-100)	100 (83.3-100)	100 (100-100)	<0.001
*p* *	-	0.010	0.004	-
MH	60 (48-76)	72 (56-80)	76 (56-86)	0.009
*p* *	-	0.366	0.010	-
PCS	38.6 (32.8-44.8)	53.8 (47.7-57.3)	56.1 (51.9-59.1)	<0.001
*p* *	-	<0.001	<0.001	-
MCS	40 (29.5-51.1)	54 (47.7-56.9)	53 (45.5-59)	0.001
*p* *	-	0.009	0.001	-

*p* value^#^ = *p* value was determined by the Friedman test comparing changes in scores along the follow-up.

*p* value* = *p* value was determined by the Wilcoxon signed-rank test comparing the 6-mo scores and 12-mo scores to the baseline values.

HRQoL = health-related quality of life; IQR = interquartile range; PF = physical function; RP = role-physical; BP = bodily pain; GH = general health; VT = vitality; SF = social functioning; RE = role-emotional; MH = mental health; PCS = physical component summary; MCS = mental component summary.

Regarding the baseline HADS-anxiety and HADS-depression, 28 (68.3%) and 31 (75.6%) patients had anxiety and depression symptoms, respectively. However, the incidence was not significant decreased at 6 months and 12 months postoperatively when comparing to the baseline (Supplementary Figure-1).

Compared with preoperative scores, there were statistically significant modifications in overall OHIP-14 and functional limitation after surgery (Table-S2). However, no statistically significant difference was found when comparing the baseline values to those at 6 months and 12 months postoperatively in all domains (p < 0.05), including overall OHIP-14, functional limitation, physical pain, psychological discomfort, physical disability, psychological disability, social disability, and handicap.

## DISCUSSION

Given the successful experiences with various materials in urethral reconstruction(13, 14), oral mucosal grafts have been increasingly utilized by urologists to facilitate the management of complex proximal or middle US. In 2015, Li et al. (1) reported the first case of LMGU for complex US and achieved satisfactory outcomes Since then, this procedure has gained popularity and several studies have reported excellent results of LMGU (3, 4, 7-9). However, the longitudinal changes of patient-reported outcomes remained elusive. In this study, we prospectively included 41 patients with complex US undergoing minimally invasive LMGU, to evaluate objective treatment efficacy and safety, as well as subjective patient-reported outcomes, including HRQoL, mental health, and OHRQoL. Consistent with previous reports, minimally invasive LMGU achieved a success rate of 97.56% (40/41) during the median follow-up of 29 (range 15-41) months. There were no major postoperative complications (grade III and IV); there was only 7 (17.07%) patients experiencing minor complications (grade I and II). There results suggested that minimally invasive LMGU is a safe, feasible and efficient procedure for complex ureteral reconstruction.

One of the most important goals of any surgical interventions is to improve patients’ quality of life. Although the technical nuances and postoperative morbidity have been well documented (3, 4, 9), there is no available data regarding the measurement of longitudinal changes in HRQoL associated with ureteral reconstruction using LMG. Similar to previous reports (15), our findings revealed that patients with complex US presented significantly decreased HRQoL compared with the general population. Encouragingly, patient reported HRQoL significantly improved at 6 months and 12 months following LMGU. Most patients achieved HRQoL success in PCS and MCS at 12 months postoperatively. These satisfactory results could be linked to the surgical outcomes of alleviated symptoms and avoided long-term indwelling nephrostomy tube or ureteral stents. Thus, HRQoL assessments should be more routinely used to assess the effect of ureteral reconstruction, identify patients who might benefit from surgery, and justify the efforts and expense of health care teams caring for patients with complex US.

Surprisingly, the improvement of patient-reported mental health was comprised following LMGU. According to our results, only 63.4% of patients achieved HRQoL success in MCS at 12 months postoperatively. Moreover, there was no significant reduction in patients’ anxiety and depression levels during the follow-up period, despite the high prevalence of patients with anxiety and depression symptoms. This could be attributed to the negative illness perceptions and concerns about renal impairment. Therefore, timely and appropriate psychological interventions should be provided for patients with poor mental health for better health care outcomes. Future research with larger sample size and longer follow-up period will further illuminate the underlying causes for the comprised efficacy of LMGU in alleviating patients’ anxiety and depression.

While preserving renal function, LMGU would inevitably bring surgical trauma to the tongue. In our study, all patients returned to a regular diet within a few days after surgery. Only four patients reported numbness of the tongue within 1 month after surgery. In the series of LMGU (n = 41) with a median follow-up of 35 months, Liang et al. reported that no patients experienced oral-related complications such as severe pain in tongue, difficulty in mouth opening and loss of taste (4), which is consistent with our results. In this study, we firstly used an OHIP-14 survey to longitudinal evaluate patient-reported OHRQoL following LMGU. Our findings confirmed that the OHRQoL returned to the baseline level at 6 months and 12 months postoperatively, despite the early trauma to tongue. These results suggested that the surgical damage to the tongue is reversible with minor discomfort for patients. Additionally, the recovery of OHRQoL proved to be irrelevant with the length of the tongue mucosa harvested during the operation, indicating that longer lingual mucosa could be considered if necessary.

It has been reported that the donor site morbidities for buccal mucosal graft (BMG) were mostly comprised by oral numbness (16%), difficulty in mouth opening (32%) and salivary function changes (16-18). In our study, fewer donor site morbidities were observed for LMG compared with BMG, which has formerly been validated in urethroplasty. Kumar et al. reported that LMG urethroplasty achieved a similar clinical success rate to BMG urethroplasty with lower donor site morbidity (19). Moreover, LMG is easier to harvest than BMG, particularly for patients with small open mouths (20). Thus, LMG is considered as an ideal choice for ureteroplasty, while further studies are still needed to compare the efficacy and safety between these two procedures.

Several limitations exist for this study. Firstly, the sample size is relatively small, thereby weakening our conclusions. Second, OHIP-14, the questionnaire used to assess patients’ OHRQoL, is unable to directly measure the impact of surgery on tongue functions, such as speech, deglutition, tongue mobility, etc. Further studies should prospectively evaluate the direct effects. Finally, the 12-months follow-up may be insufficient to evaluate the long-term impact of both disease and treatments on HRQoL. Our study protocol, however, includes a 24-months evaluation of the HRQoL, but the collection of the 24-months data is ongoing and will be the subject of future reports.

## CONCLUSIONS

LMGU is a safe and efficient procedure for complex ureteral reconstruction that significantly improves patient reported HRQoL without compromising OHRQoL. Assessing patients’ quality of life enables us to monitor postoperative recovery and progress, which should be considered as one of the criteria for surgical success.

## Figures and Tables

**Figure:**
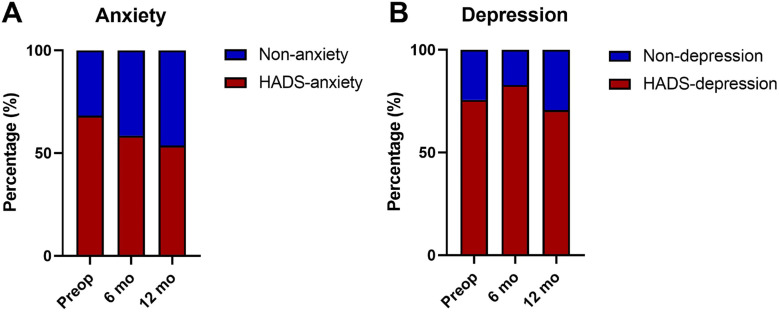
Longitudinal changes of HADS-anxiety and HADS-depression. (A) The percentage of HADS-anxiety and non-anxiety over time. (B) The percentage of HADS-depression and non-depression over time.

## Data Availability

The data that support the findings of this study are available from the corresponding author upon reasonable request.

## Abbreviations

US =: ureteral stricture
LMGU =: lingual mucosal graft ureteroplasty
HRQoL =: health-related quality of life
OHRQoL =: oral health-related quality of life
LMG =: lingual mucosal graft
STROBE =: Strengthening the Reporting of Observational Studies in Epidemiology
RECUTTER =: Reconstruction of Urinary Tract: Technology, Epidemiology and Result
SF-36 =: MOS 36-item health survey
HADS =: hospital anxiety and depression scale
OHIP-14 =: Oral Health Impact Profile-14
PF =: physical function
RP =: role-physical
BP =: bodily pain
GH =: general health
VT =: vitality
SF =: social functioning
RE =: role-emotional
MH =: mental health
PCS =: physical component summary
MCS =: mental component summary
SD =: standard deviation

## APPENDIX:

### Supplementary figure 1

**Supplementary Table 1:** Comparison of preoperative HRQoL scores in patients with complex US (n = 41) and Chinese general population (n = 3214).

Dimension	Mean study population ± SD	Mean general population ± SD	*p* value (*t* test)
PF	74.15 ± 18.16	94.02±12.44	<0.001
RP	21.95 ± 34.55	88.79±28.49	<0.001
BP	64.85 ± 16.36	88.18±19.02	<0.001
GH	56.10 ± 19.39	69.74±20.95	<0.001
VT	57.80 ± 17.64	68.92±18.78	<0.001
SF	56.40 ± 24.39	88.03±16.00	<0.001
RE	46.34 ± 42.74	89.57±27.95	<0.001
MH	62.63 ± 16.80	77.61±15.85	<0.001

HRQoL = health-related quality of life; US = ureteral stricture; SD = standard deviation; PF = physical function; RP = role-physical; BP = bodily pain; GH = general health; VT = vitality; SF = social functioning; RE = role-emotional; MH = mental health

**Supplementary Table 2:** OHRQoL scores variation in the different domains during the follow-up (mean, median and [IQR]).

Variable	Baseline	6 mo	12 mo	*p* #
OHIP-14	2.95, 1 (0-4)	4.73, 1 (0-5)	3.73, 1 (0-3)	0.013
*p* *	-	0.181	1.000	
Functional limitation	0.49, 0 (0-1)	1.02, 0 (0-2)	0.85, 0 (0-1.5)	0.035
*p* value*	-	0.408	0.739	
Physical pain	0.98, 0 (0-2)	1.17, 1 (0-2)	1.02, 1 (0-1)	0.322
*p* value*	-	-	-	
Psychological discomfort	0.32, 0 (0-0)	0.68, 0 (0-0.5)	0.46, 0 (0-0)	0.099
*p* value*	-	-	-	
Physical disability	0.51, 0 (0-0.5)	0.54, 0 (0-0.5)	0.51, 0 (0-0)	0.879
*p* value*	-	-	-	
Psychological disability	0.34, 0 (0-0)	0.56, 0 (0-0)	0.34, 0 (0-0)	0.086
*p* value*	-	-	-	
Social disability	0.29, 0 (0-0)	0.37, 0 (0-0)	0.27, 0 (0-0)	0.738
*p* value*	-	-	-	
Handicap	0.15, 0 (0-0)	0.39, 0 (0-0)	0.27, 0 (0-0)	0.069
*p* value*	-	-	-	

*p* value^#^ = *p* value was determined by the Friedman test comparing changes in scores along the follow-up.

*p* value* = *p* value was determined by the Wilcoxon signed-rank test comparing the 6-mo scores and 12-mo scores to the baseline values.

OHRQoL = oral health-related quality of life; IQR = interquartile range
